# Effect of Choline Chloride-Based DES on the Pore-Forming Ability and Properties of PVDF Membranes Prepared with Triethyl Phosphate as Green Solvent

**DOI:** 10.3390/polym17070984

**Published:** 2025-04-04

**Authors:** Alejandro Gálvez-Subiela, Ramón Jiménez-Robles, Jose David Badia-Valiente, Marta Izquierdo, Amparo Chafer

**Affiliations:** Research Group in Materials Technology and Sustainability (MATS), Department of Chemical Engineering, School of Engineering, University of Valencia, Avda. Universitat s/n, 46100 Burjassot, Spain; alejandro.galvez@uv.es (A.G.-S.); ramon.jimenez@uv.es (R.J.-R.); jose.badia@uv.es (J.D.B.-V.)

**Keywords:** poly(vinylidene fluoride) PVDF, deep eutectic solvent (DES), choline chloride, glycerol, polyethylene glycol (PEG), membrane, non-solvent induced phase separation (NIPS), green solvent, triethyl phosphate (TEP)

## Abstract

This study explores the influence of various additives on the morphological, chemical, and thermal properties of poly(vinylidene fluoride) (PVDF) membranes prepared via the non-solvent induced phase separation (NIPS) technique. The use of a green solvent such as triethyl phosphate (TEP) was shown to be successful. A particular focus was dedicated to pore formers based on choline chloride–based deep eutectic solvents (DES) in combination with ethylene glycol and glycerol, i.e., ChCl/EG and ChCl/GLY, and its benchmark with traditional counterparts such as poly(ethylene glycol) (PEG) and glycerol (GLY). Comprehensive characterization was conducted using FESEM, FTIR, XRD, and DSC techniques to evaluate changes in membrane morphology, porosity, and crystallinity. PEG acted as a pore-forming agent, transitioning the internal structure from spherulitic to sponge-like with consistent pore sizes, while GLY produced a nodular morphology at higher concentrations due to increased dope solution viscosity. DES induced significant shifts in crystalline phase composition, decreasing α-phase fractions and promoting β-phase formation at higher concentrations. While the overall porosity remained unaffected by the addition of GLY or PEG, it was dependent on the DES concentration in the dope at lower values than those obtained by GLY and PEG. Membrane pore size with ChCl/GLY was lower than with ChCl/EG and GLY. All membranes showed performance at the hydrophobic regime. The findings demonstrate that ChCl/EG and ChCl/GLY can tailor the structural and thermal properties of TEP-driven PVDF membranes, providing a green and versatile approach to customize the membrane properties for specific applications.

## 1. Introduction

Global warming and climate change pose pressing environmental and social challenges, driven primarily by high levels of greenhouse gas emissions from the transportation, energy production plant, and industrial sectors [1]. To partially mitigate its effects, the development of sustainable technologies for CO_2_ capture from industrial emissions has gained paramount importance [2,3]. In this sense, membrane technology provides a straightforward, scalable, and energy-efficient alternative to traditional gas separation methods such as chemical absorption [4], adsorption [5], and cryogenic distillation [6], which tend to have a very high energy-intensiveness [7]. Membrane technology for CO_2_ capture can be applied in gas-liquid systems contactors, in which the CO_2_ is transferred to a liquid absorbent with a hydrophobic membrane acting as a physical barrier between the gas and the liquid absorbent. The gas-liquid membrane contactors have also been applied for dissolved CH_4_ recovery from effluents from anaerobic digesters [8,9] to mitigate the CH_4_ fugitive emissions when these effluents are discharged.

Membrane technology offers high versatility in material properties, different configurations, and variety in target gas species; thus, high interest in this technology exists [9], [10,11,12]. A common method for fabricating membranes is the non-solvent induced phase separation (NIPS) technique, which enables control over membrane morphology and performance through careful selection of solvents, additives, and processing conditions [13,14]. The choice of polymer solvents in the NIPS process plays a critical role in membrane characteristics [15]. Triethyl phosphate (TEP) has emerged as a promising alternative to conventional polymer solvents such as NMP, DMAc, or DMF, and the main advantages of its use are the low toxicity and hazards for workers in membrane manufacturing at the industrial level; in addition, due to its high boiling point (215 °C), it has lower volatility. In addition, TEP shows a lower polymer-solvent distance than conventional solvents when it refers to PVDF, which generates a high interest in its use. These properties contribute to TEP’s potential as a more environmentally friendly alternative to solvents currently used in the industry [16,17]. However, its use with NIPS-driven PVDF preparation is not yet well stablished due to its high kinetics of the coagulation process [18]. In addition, poly(ethylene glycol) (PEG) and glycerol (GLY) are commonly used to modify mechanical strength, porosity, and surface qualities in membranes [19,20,21]. As a pore-forming agent, PEG is frequently employed to promote connected pore networks that improve membrane permeability [22,23]. GLY is a common plasticizer that assists in the polymer matrix becoming more flexible and less brittle, characterized by generating changes in membrane morphology, such as the pore size of the material [24]. However, interest in alternatives has increased due to concerns about the impact of these usual chemicals on the environment [25].

Deep eutectic solvents (DESs) have emerged as a transformative innovation in membrane technology as dope additives in the NIPS process [26]. DESs are synthesized through interactions between hydrogen bond donors (HBDs) and acceptors (HBAs), resulting in a typically wide array of properties such as low expense, biodegradability, and non-toxicity [27,28]. DESs are distinguished by their exceptional adaptability, as their combination of multiple HBDs and HBAs enable precise adjustment of the additives’ physical and chemical properties [29]. This versatility has focused efforts on the study and possible implementation of DESs as additives or modifiers in the preparation of membranes [30,31,32]. In many cases, the use of DESs in the manufacture of membranes has generated interesting changes in the properties of the material, such as increases in porosity, modifications in pore size or roughness, or changes in morphology. For example, Kumar et al. (2021) used a 1.0 wt% of a DES based on choline chloride/urea at different ratios (1:2, 1:3, 1:4, 1:5) as an additive to the dope solution in the fabrication of polyethersulfone ultrafiltration membrane using DMSO as the solvent, and increases in the membrane porosity and mean pore size were detected [33]. Chen et al. (2023), using PVDF as a polymer, DMAc as a solvent, and the hydrophilic DES based on betaine/lactic acid (1:2) in the coagulation bath at a concentration from 1.0 to 20 wt%, observed an increase in the membrane porosity and the formation of finger-like macrovoids at high DES concentrations [34]. Elhamarnah et al. (2023) obtained an increase in the ultrafiltration membrane porosity by adding a DES consisting of choline chloride (ChCl) and fructose (1:1) at 4 wt% concentration in the polysulfone polymeric solution using NMP as a solvent [35]. Likewise, Yeow et al. (2024) presented an extensive study of the formation of PVDF membranes using DMF as a solvent with five ChCl-based DESs using urea, glycerol, ZnCl_2_, lactic acid, or glucose as the HBD at a concentration of 2 wt%, observing relevant modifications in the internal structure of the membranes though losing tensile strength for several membrane formulations with DESs [36]. Most of the studies are based on the use of conventional solvents, and the combined use of TEP as a solvent and a DES as a dope additive is scarcely studied.

Among the various DES-forming compounds, DESs formed by ChCl present exceptional solvation capability, a wide possibility of combinations with HBDs, and high polarity, which is expected to produce important changes during membrane formation in the NIPS method, accelerating the coagulation process [34]. ChCl-based DESs, considered type III DESs, are compounds with low cost, low toxicity, biodegradability, and biocompatibility, resulting in an easy preparation method with a wide availability of the primary resources [26,37]. Likewise, the use of DESs, particularly those based on ChCl, has shown promising potential in terms of cost-effectiveness in different applications [38,39]. In addition, the ability of ChCl-based DESs as dope polymer additives to promote relevant morphological changes has been demonstrated in several previous studies [33,35,36,40,41]. All these characteristics make the ChCl-based DES a prime candidate for advancing in membrane technology.

Poly(vinylidene fluoride) (PVDF) is a prominent polymer in membrane applications due to its outstanding chemical resistance, mechanical strength, and thermal stability [42,43]. These properties, together with the ease of surface functionalization, make PVDF membranes suitable for a wide range of demanding applications, including harsh chemical environments and high-temperature conditions [44,45,46]. However, the manufacturing process for PVDF membranes traditionally relies on solvents (N-methyl-2-pyrrolidone, dimethylformamide, or dimethylacetamide) and additives that can pose environmental and health risks [16]. Triethyl phosphate enables effective phase separation in the fabrication of PVDF membranes and aligns with the growing emphasis on green chemistry principles [17,47]. Its lower toxicity and environmental impact make it a suitable choice for researchers aiming to reduce the ecological footprint of membrane production [17,48].

The present study explores the effect of incorporating several additives, including two different DESs as novel additives, to the PVDF dope solution on the morphological, chemical, and thermal characteristics of the resulting PVDF membranes fabricated by NIPS. In particular, the effect of two different HBDs (ethylene glycol and glycerol) with chlorine-chloride as an HBA was investigated. The effect of increasing the concentration of these additives was studied and compared with poly(ethylene glycol) and glycerol as common additives. Changes in morphology, overall porosity, pore size, thickness, hydrophobicity, surface chemical composition, crystallinity, and thermal properties were analyzed.

## 2. Materials and Methods

### 2.1. Materials

Powder PVDF with a density of 1.76 kg/m^3^ and melt viscosity from 23.50 to 29.50 kP (poly(vinylidene fluoride), Thermo Scientific Chemicals, Waltham, MA, USA) was used as a polymer matrix. Triethyl phosphate (TEP; >99.0%, TCI America, Portland, OR, USA) was chosen as a non-toxic solvent. Choline chloride (ChCl; 99%, Thermo Scientific Chemicals, MA, USA) was employed as an HBA to form hydrophilic deep eutectic solvents (DESs) with glycerol (GLY; ACS Reagent, ≥99.5, Sigma-Aldrich, Saint Louis, MO, USA) or ethylene glycol (EG; ≥99.7%, Merck, VWR Chemicals, Fontenay-sous-Bois, France) as an HBD. Poly(ethylene glycol) (PEG; 400 g mol^−1^; GPR Rectapur^®^, VWR Chemicals, Fontenay-sous-Bois, France), glycerol, and synthesized DES (ChCl/EG and ChCl/GLY, see Section 2.2) were used as dope additives for membrane preparation. Ethanol (96%, Merck, Madrid, Spain) was used as a non-solvent for the NIPS process. Finally, 1-octanol (OcOH; 99%, Thermo Scientific Chemicals, Waltham, MA, USA) was used as impregnating liquid during overall porosity tests, and porefil^®^ (Porometer, Aptco Group, De Pinte, Belgium) was used for porometry tests.

### 2.2. Deep Eutectic Solvents Preparation

Two different DESs with ChCl as an HBA and two different HBDs (GLY and EG) were prepared to be used as membrane dope additives in flat-sheet PVDF membranes. ChCl was dried at 400 mbar and 50 °C overnight in a vacuum oven (LBX OVV, Labbox Instruments, Premià de Dalt, Spain). GLY or EG was mixed with ChCl in a 2:1 molar ratio at 60 °C under magnetic stirring at 350 rpm (MS7-H550-Pro, Onilab, Premià de Dalt, Spain), for the preparation of GLY-based DES (ChCl/GLY) or EG-based DES (ChCl/EG), respectively. After 1 h, the prepared DESs were stored at 50 °C for their future use.

### 2.3. Membrane Preparation

Flat-sheet PVDF-based membranes were prepared by solve-casting. PVDF dope solutions of 15 wt% were prepared, in which PVDF was previously dried under vacuum conditions at 400 mbar and 50 °C overnight (LBX OVV, Labbox Instruments) and stored in a desiccator. Then, dried PVDF was dissolved in TEP at 80 °C, at 200 rpm for 6 h in an orbital shaker (Rotaterm 3000435, P-SELECTA, Barcelona, Spain). The corresponding additive was then added, and the solution was stirred under the same conditions for 2 h. The solution was placed in an ultrasonic bath (Elmasonic P series, Avantor, Singer, Germany) under conditions of 80 kHz, 100% power, and 80 °C for 30 min and kept at 80 °C overnight for degassing. Additive concentrations of 1.25, 2.50, 5.00, 7.50, and 10.00 wt% were tested for the membrane dope formulations.

The viscosity of the prepared dope solutions was measured by viscometer equipment (Brookfield Programmable DV-II+ Viscometer, Brookfield Engineering Labs., Inc., Middleboro, MA, USA) using the spindle #1 (LV Spindle set, Brookfield Engineering Labs., Inc., Middleboro, MA, USA). The sample volume was 30 mL, collected at 80 °C and measured for 2.5 rpm rotation velocity, and the results are presented in Table 1.

Membranes from dope solutions were casted with a film applicator (Automatic Film Applicator Standard Heated Vacuum, TQC Sheen, Industrial Physics Inc., Capelle aan den IJssel, The Netherlands) at 80 °C, using a casting knife (Dr. Blade, BYK, Geretsried, Germany) with a fixed height of 800 µm and constant speed of 20 mm/s, onto a 20 × 20 cm^2^ glass. The polymeric film on the supporting glass was kept in a 0.5 L ethanol bath for 3 h at room temperature (~24 °C) to produce the membrane coagulation of the NIPS process. Then, the upper side of the membrane was washed with distilled water. The membrane was placed into a drying chamber with a synthetic air flow rate of 0.5 L h^−1^ overnight. The membrane was impregnated with 2-propanol (99.9%, Labbox Labware, S.L., Barcelona, Spain) to detached it from the glass, placed on filter paper, and dried for 2 h in the drying chamber under the same conditions.

### 2.4. Analytical Techniques for Membranes Characterisation

The surface and cross-section morphology of membranes were analyzed by field emission scanning electron microscopy (FESEM) with an accelerating voltage of 5 kV (Hitachi S4800, Hitachi, Ltd., Tokyo, Japan). For the image acquisition, the samples were placed on a metal holder and then coated with a layer of Au/Pd by sputtering in a vacuum for 40 s.

Overall porosity was measured using a gravimetric method [49], using OcOH as an impregnation solvent. Membrane coupons with a size of 2 × 2 cm^2^ were weighed (HCB 1002 Highland, Adam equipment, Milton Keyne, UK) before their immersion in 10 mL of OcOH and agitation for 2 h at room temperature (~24 °C) and 100 rpm in an orbital shaker (Orbital Shaker Orb-b2, LBX OVV, Labbox Instruments, Premià de Dalt, Spain). Later, the excess of the solvent was removed, and specimens were weighed again. Equation (1) was used to determine the membrane specimen overall porosity:

(1) $$\varepsilon =\frac{m_{wet}-m_{dry}}{\rho_{solv}\cdot A\cdot \delta }\cdot 100$$

where m_wet_ (kg) and m_dry_ (kg) are the masses of the wet and dry specimens, respectively, ρ_solv_ (kg m^−3^) is the density of 1-octanol, and A (m^2^) and δ (m) are the area and thickness of the specimen, respectively.

The membrane thickness (δ) was measured using a digital micrometer (Digital Indicator ID-C, Mitutoyo, Dusseldorf, Germany). Results are expressed as the average and standard deviation of at least 5 specimens.

The pore size distribution was measured by capillary flow porometry according to the ASTM F316-03 standard [50], and the pore size diameter (D_p_, µm) is reported. Porefil^®^ was chosen as the wetting liquid. A 25 mm diameter membrane sample was immersed in 20 mL of wetting liquid and kept at 100 rpm in an orbital shaker for 2 h at room temperature (~24 °C). A digital manometer (MD680, Melven, Sant Boi de Llobregat, Spain) was used to measure the air pressure applied (∆P), and a gas flowmeter (Whisper MW/MWB-Series, Alicat, Duiven, The Netherlands) was used to measure the air flow rate. Then, continuous values of pressure and airflow, using the flow meter (Whisper MW/MWB-Series, Alicat, Duiven, The Netherlands), were collected until they completely dried off the membranes. Washburn’s equation (Equation (2)) was used to calculate the membrane’s pore size.

(2) $$D_{p}=\frac{4\cdot \gamma \cdot \cos\theta \cdot SF}{∆P}$$

where D_p_ is the pore size diameter (µm); γ is the surface tension of the Porefil^®^ (0.01637 N m^−1^ at 20 °C); θ is the contact angle of the compound used with the sample, with zero contact angle for Porefil^®^ [51]; SF is the shape factor (0.715, assuming the presence of elliptical pores [51]); and P is the pressure (bar) of the compressed air.

The water contact angle (WCA) was measured with the sessile drop technique to evaluate the surface hydrophobicity of the membranes. An amount of 5.5 ± 0.1 µL of water was deposited on the membrane surface with a syringe pump (KF Technology s.r.l., Rome, Italy). An image of the water drop profile was captured at 15 s with a digital microscope (Handheld Digital Microscope Pro, Celestron LLC., Torrance, CA, USA) illuminated by a white light (Philips HUE Lamp, Koninklijke Philips NV, Amsterdam, The Netherlands). ImageJ software, 1.54 g version, was used to process the images, using the Contact Angle Plug-in based on the ellipse approximation. Results are reported as the mean values and standard deviations from at least 4 measurements of 2 specimens.

The melting and crystallization temperatures along with their respective heat flux were determined using differential scanning calorimetry (DSC; Setline^®^, Setaram Kep Technologies, Caluire-et-Cuire, France). All analyses were carried out using ~4 mg of the sample. The analyses were conducted at a heating rate of 10 °C min^−1^ from 60 to 210 °C and a cooling rate of 10 °C min^−1^ to the initial temperature, under a nitrogen atmosphere with a flow rate of 50 mL min^−1^, with a minimum of 2 replicates for each membrane.

The Fourier transform-infrared spectroscopy spectra (FTIR) in the attenuated total reflectance mode were recorded (Cary 630 FTIR Spectrometer, Agilent Technologies, Inc., Santa Clara, CA, USA), in the 4000–500 cm^−1^ range and a resolution of 4 cm^−1^, and processed with the Agilent Microlab FTIR software, MicroLab v5.7 version. From FTIR results, the mole fractions of the α and β crystalline phases in each of the membranes (x and y, respectively) were calculated with Equation (3) and assuming that x + y = 1:

(3) $$y=\frac{A_{\beta }}{\left(\frac{K_{\beta }}{K_{\alpha }}\right)A_{\alpha }+A_{\beta }}\times 100$$

where y represents the mole fraction of the β-crystalline phase, A_α_ and A_β_ represent absorbance levels at the wavelengths of 766 cm^−1^ and 840 cm^−1^, respectively, from the infrared spectra, and K_α_ (6.1 × 104 cm^2^ mol^−1^) and K_β_ (7.7 × 104 cm^2^ mol^−1^) are the absorption coefficients of the α and β phases, respectively [52]. The absorbance values for each crystalline phase were quantified from the FTIR analyses subsequently presented. Then, the total degree of crystallinity (X, %) of the prepared membranes was calculated with Equation (4):

(4) $$X=\frac{{\Delta h}_{m}}{x{\Delta h}_{\alpha }+y{\Delta h}_{\beta }}\times 100$$

where ∆h_m_ (J g^−1^) is the experimental melting enthalpy of the sample obtained from the differential scanning calorimetry analysis, ∆h_α_ (93.07 J g^−1^) and ∆h_β_ (103.40 J g^−1^) are the melting enthalpies of a 100% crystalline PVDF in the α and β phases [53], respectively, and x and y are the molar fractions of the α and β phases in the sample, respectively, which were assumed to be the predominant phases (x + y = 1), obtained from the FTIR results.

X-ray diffraction (XRD) analysis was carried out for the identification of crystalline phases in the solid particles formed in the crystallization tests. The diffractograms were obtained with an XRD system (AXS D8 ADVANCE A25, Bruker, Billerica, MA, USA).

## 3. Results and Discussion

The effect of the use of two DESs as dope additives (ChCl/EG or ChCl/GLY) on the PVDF membrane properties is compared with the use of PEG or GLY. The tested range of additive concentration was 0–10 wt% for all the cases. Results are discussed in this section in terms of morphological and surface properties, chemical and crystallinity analyses, and thermal properties.

### 3.1. Effect of Additives on Morphology of Membranes

FESEM images were taken for inspection of the surface and cross-section of the PVDF membranes, and the results are shown in Table 2. A limit on the maximum concentration of additives was found for the use of ChCl/EG, ChCl/GLY, and GLY. Concentrations >10 wt% of ChCl/EG and GLY in the dope solution and ≥7.5 wt% of ChCl/GLY resulted in PVDF membranes with poor mechanical integrity, and results of membrane characterization were not performed. Previous studies where PVDF membranes have been fabricated with a DES as an additive have not presented concentrations higher than 5.00 wt% [36,54]. This agrees with the loss of consistency shown by the membranes at high concentrations of DESs in the present study.

For the pristine PVDF membrane, a top porous layer and a spherulitic structure along the cross-section were observed (Table 2). The appearance of this morphology inside the membrane is typical of a slow mass transfer in the liquid-liquid demixing process [55]. Both the membrane’s internal structure and the surface images showed a highly porous material, confirmed with overall porosity results close to 80% in most cases (Table 3).

The addition of PEG at different concentrations resulted in an overall porosity without significant variations, providing values close to 80% for the pristine PVDF membrane and membranes with PEG at all tested concentrations. These values are similar to other studies where PEG was used as a pore former in the preparation of flat sheet membranes, such as Gayatri et al. (2023), who presented overall porosity values of 78% for PVDF-PEG membranes [56]. However, the addition of PEG in the membrane formulation generated a significant change in the internal structure of the membrane, as shown in the cross-section images (Table 2) independently of the PEG concentration. The addition of PEG resulted in a change in the internal morphology from a spherulitic structure to a sponge-like structure. The obtained structure was similar to that shown in a study by Zhao et al. (2007) where PEG was added to the dope solution, using N, N-dimethylacetamide as a solvent and PEG with a molecular weight of 5500 g mol^−1^ [57]. The generation of an internal sponge-like structure is linked to the slowing down of the polymer coagulation process since rapid coagulation processes promote the formation of macro voids and finger-like structures inside the membrane [58]. The increase in the dope solution viscosity with the PEG as an additive could also have been the cause of the slower rate formation of the solid phase in the nascent membrane. Regarding the thickness of the fabricated membranes (Table 3), it can be observed that with PEG, the thickness was similar to that of the pristine PVDF membrane, with final thickness values close to 290 µm. The fact that average values of the overall porosity or thickness of the membranes prepared with PEG did not suffer a decrease suggested that the PEG could work as a pore-former in the membrane, since the PEG prevented the collapse of the pores formed during polymer coagulation [22]. This could explain why the thickness was not affected by the addition of PEG to the dope solution.

Results of the most probable pore size (D_p_) of each of the fabricated membranes are presented in Table 3, along with the pristine PVDF without an additive. The D_p_ of the pristine PVDF was 3.30 µm, similar to that reported by Lin et al. (2006), who concluded that the large pore size obtained in PVDF membranes between 2 and 3 µm could be due to the use of a very soft bath of distilled water [59]. The porometry test results presented showed that the D_p_ in the membranes with PEG were very close to each other regardless of the PEG concentration, with values between 0.66 and 0.77 µm, indicating that the sponge-like structure of these membranes had pores of similar size. Moreover, these D_p_ values were much lower than that of the pristine PVDF membrane (3.30 µm). Other authors also reported the reduction in pore size with the use of PEG, with pore sizes close to 1 µm reported for PEG at low concentrations between 0.2 and 3.0 wt% [23]. The reduction in the D_p_ with the addition of PEG was attributed to the morphological change observed in the SEM images [22], since the change from a spherulitic structure to a sponge-like structure involved the compaction of the bulk material, resulting in lower pore sizes.

Regarding the use of ChCl/EG as an additive, the resulting membranes showed small differences in their surface and cross-section (Table 2), maintaining the pristine membrane’s spherulitic structure but with the appearance of an increased number of nodules along the cross-section. An increase in voids or channels in the bulk of the membranes was observed with the increased ChCl/EG concentration, becoming noticeable especially for ≥5.00 wt% of additive content. In addition, an increase in the membrane pore size was observed (Table 3). While the pristine PVDF membrane gave D_p_ results of 3.30 µm, with the addition of ChCl/EG, values above the detection limit of the test (>6.60 µm) were reached in some samples. The presence of large pore size indicated that the introduction of an additive of solvent nature, unlike the PEG previously studied, resulted in noticeable structural changes. These results could suggest an affinity between the ChCl/EG additive and the non-solvent, i.e., ethanol, promoting the acceleration of the liquid-liquid demixing process during the formation of the membrane, resulting in large pore sizes [60].

The thickness of the membranes was significantly reduced by the addition of ChCl/EG to the polymer dope, in comparison to pristine PVDF and to membranes with PEG (Table 3). The PVDF pristine membrane presented a value close to 300 µm; however, with the use of ChCl/EG, the thickness resulted in values around 240 µm. Thickness reduction was also seen by Fang et al. (2023) in earlier studies using ChCl/EG as a co-solvent in polyether sulfone membranes [61]. Regarding the overall porosity of ChCl/EG membranes, a decrease was observed from 80.4% without additive to around 75% for ChCl/EG concentration ≥ 5.0 wt%, while the surface density remained in values around 10 mg cm^−2^. The reduction in thickness and overall porosity was attributed to the affinity with the non-solvent previously stated. Thus, while the PEG additive acted as an impediment to the collapse of the internal porosity of the membranes during their formation, the solvent nature of ChCl/EG resulted in the outflow of the additive from the polymer matrix.

Regarding the use of GLY as an additive, FESEM images shown in Table 2 revealed that the cross-section of the membranes suffered important changes as the GLY concentration increased. The minor concentration studied, i.e., 1.25 wt%, showed a change in the internal structure of the membranes from a spherulitic morphology to a sponge-like structure. The structure of the membranes with higher GLY content, between 2.50 and 7.50 wt%, seems to present a nodule morphology in their cross-section, and this was attributed to a higher speed in the liquid-liquid demixing process of the NIPS method. A similar structure was previously reported with the use of GLY and lithium chloride as an additive [60] and with the use of GLY in PVDF membranes, using water as a coagulation medium [62]. The increase in viscosity of the polymer dope with the increase in GLY (Table 1) can be the cause of the changes in the membrane morphology [63], resulting in an intermediate structure between sponge and spherulitic shapes, called a nodule structure. This viscosity increase was related to the formation of bridging complexes between the GLY, the polymer fluorine, and the solvent, which deteriorated the flexibility of the polymer chains, causing a decrease in the distributive freedom of the polymer in the dope solution [64].

The overall porosity slightly increased by the addition of GLY to the dope solution with respect to the pristine membrane, with an overall value around 82% (Table 3). Likewise, the thickness of the membranes with GLY presented similar values independently of its concentration, with thicknesses between 300 and 320 µm and close to 294.2 ± 16.5 µm of the pristine PVDF membrane. These results indicated that the use of GLY as an additive acted as a pore former, as with the use of PEG. In this sense, the addition of GLY affected the values of D_p_ (Table 3). The use of low GLY concentration (1.25 wt%.) reduced the pore size to D_p_ with respect to the pristine PVDF membrane (D_p_ = 3.30 µm), with a value of 2.31 µm. The increase of GLY to 2.50 and 5.00 wt% showed a D_p_ higher than the pristine PVDF membrane, with values of 3.85 and 3.55 µm, respectively. The increase in pore size with the addition of GLY was previously reported, with an increase from 0.5 to 1.6 µm at a GLY concentration from 1 wt% to 4 wt% [24,65]. Further increase in the concentration of GLY to 7.50 wt% resulted in the decrease of the pore size (D_p_ = 2.01 µm). The high viscosity of the polymer dope solution with 7.5 wt% GLY (Table 1) may have generated a slower diffusion between the solvent and the non-solvent in the NIPS process, resulting in the diminution of the pore size with respect to the lower GLY concentrations and causing the internal membrane structure to approach a sponge-like morphology.

Regarding the use of ChCl/GLY, while the morphology of the membranes’ surfaces from SEM images did not show major differences compared to the rest of the additives, changes in their cross-section are noticeable (Table 2). A nodular structure was observed in the cross-section of the membrane, similar to the one observed with the use of ChCl/EG at 2.50 and 5.00 wt%. Concerning the overall porosity, thickness, and surface density, results were in agreement with the use of ChCl/EG (Table 3), with a reduction in overall porosity and thickness with the increase in ChCl/GLY concentration and with an almost constant surface density. Again, the DES compound of ChCl/GLY did not act similarly to its counterpart GLY, which prevents the collapse of the internal structure and maintains the membrane’s internal structure during its formation. Regarding the pore size, the use of ChCl/GLY resulted in lower pore size than the pristine PVDF for the lowest concentrations, with D_p_ values of 2.10 and 2.72 for 1.25 and 2.50 wt%, respectively. Thus, ChCl/GLY could have an influence on the NIPS process by decreasing the rate of membrane formation, obtaining smaller pore sizes at low DES concentrations than in the pristine PVDF membrane. However, further increase in the ChCl/GLY resulted in big pore sizes above the detection limit, indicating a threshold that should not be surpassed.

### 3.2. Influence of Additives on the Membrane’s Hydrophobicity

Hydrophobicity is another relevant design parameter in membrane preparation for gas-liquid applications [8]. Results of the water contact angle (WCA) of the pristine PVDF membrane and with the different concentrations of the additives studied are presented in Table 4. The pristine PVDF membrane presented a WCA above 140°, maintaining membranes with different additive hydrophobic values irrespectively of concentration. Comparing the results obtained with the use of PEG or ChCl/EG as an additive, for the same concentration of both compounds, it was observed that the use of a DES provided higher WCA values than PEG at all concentrations. This may be due to the solvent nature of ChCl/EG, which, unlike PEG, would have left the membrane sinus during the NIPS process. Thus, the presence of PEG in the membranes would have contributed to a more significant decrease in WCA, due to the molecular enrichment of PEG on the surface of the membranes [66]. Similar conclusions can be inferred when comparing the WCA results of GLY and ChCl/GLY. The use of ChCl/GLY resulted in higher hydrophobicity at all concentrations than with GLY. In addition to the solvent nature of DES, the increased hydrophobicity of ChCl/EG and ChCl/GLY could also be due to increased surface roughness of the membranes [67,68]. Despite the WCA variation not following a clear trend, it was clearly kept at a high hydrophobic regime at concentrations ≤5.0 wt%, which is relevant for the intended application. Consequently, FTIR, XRD, and DSC results will be analyzed and compared at 5.0 wt% concentration for all additives.

### 3.3. Effect of Additives on the Chemical Structure and Crystalline Fraction

**Figure 1:**
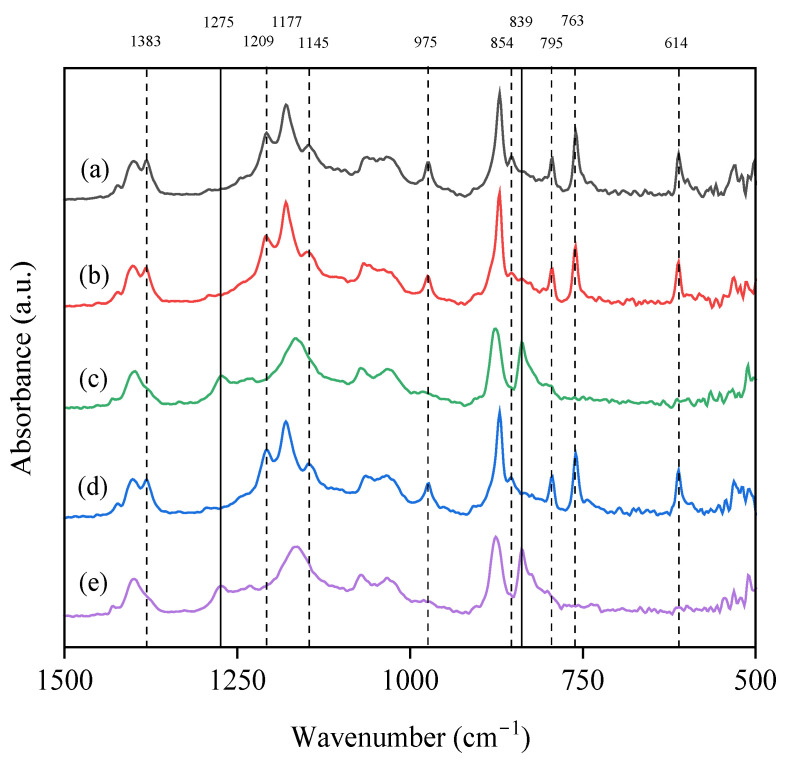
FTIR spectra for wavenumbers between 1500 and 500 cm^−1^ of the elaborated membranes with different additives at 5.00 wt%: (a) pristine PVDF membrane; (b) poly(ethylene glycol) (PEG); (c) choline chloride/ethylene glycol (1:2) DES (ChCl/EG); (d) glycerol (GLY); (e) choline chloride/glycerol (1:2) DES (ChCl/GLY). Peaks associated with α-crystalline phase bonds are in dashed lines, while the solid lines refer to β-crystalline phases.

FTIR analyses were performed on the prepared membranes, as well as on the additives used in the production of the membranes (Figure S1). The presence of PEG and GLY in the dope solutions did not cause relevant changes with respect to the pristine PVDF membrane in FTIR analysis, independently of the additive concentration, so the results are presented in Figures S2 and S3 of the Supplementary Material. However, the use of DESs as additives resulted in changes in different FTIR peaks, in the region of 500–1500 cm^−1^. Figure 1 compiles the FTIR spectra of the membranes with the four additives at the same concentration (5.0 wt%) and compared with the pristine PVDF membrane. Using DESs as additives led to a reduction in the intensity of the peaks, referring to α-phases (614, 763, 795, 854, 975, 1145, 1209 and, 1383 cm^−1^), and the appearance or increase in intensity in those corresponding to β-phases (839 and 1275 cm^−1^) [69]. The peaks related to the α and β phases did not change significantly with the use of PEG and GLY with respect to the pristine PVDF membrane spectrum (Figure 1).

**Figure 2:**
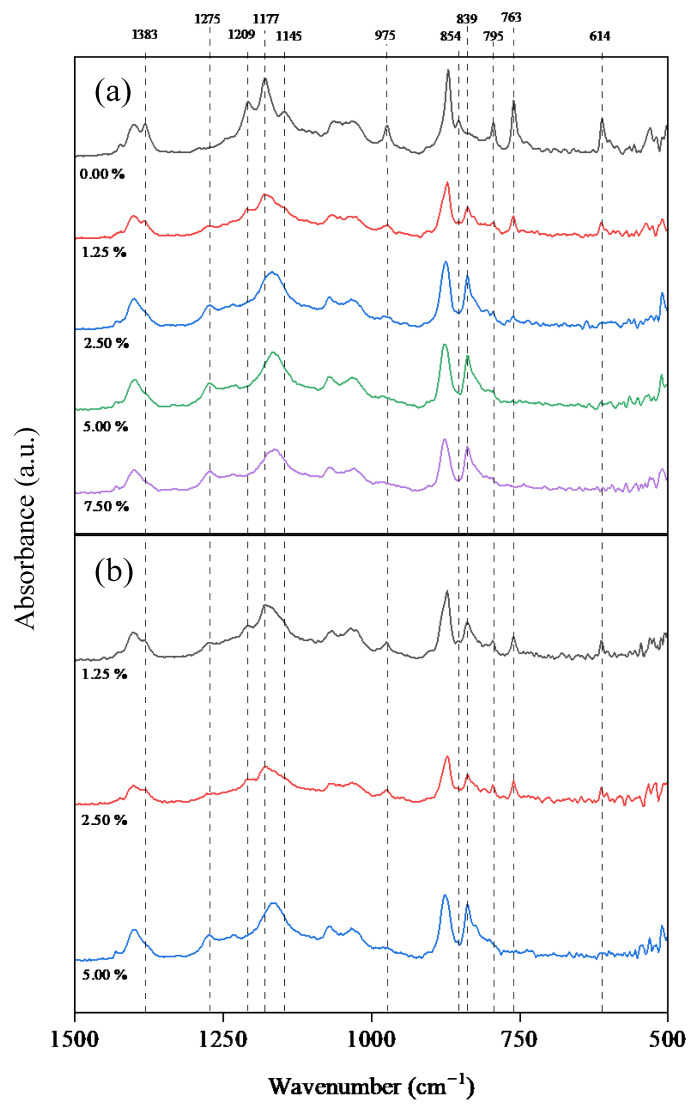
FTIR spectra between 1500 and 500 cm^−1^ of the membranes with addition of (**a**) choline chloride/ethylene glycol (1:2) DES (ChCl/EG) and (**b**) choline chloride/glycerol (1:2) DES (ChCl/GLY) at different concentrations (wt%).

For further analysis of the effect of DESs, the FTIR spectra of membranes with ChCl/EG and ChCl/GLY are presented in Figure 2 for all the studied concentrations. The changes in the FTIR peaks increased as the presence of ChCl/EG increased. The most relevant changes shown in Figure 2 corresponded to variations in the intensity of the peaks that indicate the presence of different functional groups related to α- and β-type PVDF crystalline phases. The peaks collected at 614 and 763 cm^−1^ represent the presence of fluorinated groups (C-F_2_) stretching [69], both being peaks commonly used for the quantification of α-crystalline phases in PVDF for FTIR spectra [70]. In Figure 2a, the loss of intensity at these peaks was produced with the increase in ChCl/EG concentration, almost disappearing at a concentration of 2.50 wt%. The reduction in intensity of the peak located at a wavelength of 795 cm^−1^ was also observed, corresponding to the oscillation of C-H_2_ bonds near α-crystalline structures. Conversely, there was an increase in the intensity of the peak at 839 cm^−1^ with the additive concentration, indicating an increase in C-H_2_ bonds or antisymmetric stretching of C-F_2_ bonds in β/γ-crystalline structures. A similar behavior was observed for the absorbance peak at 1275 cm^−1^, where the increase in ChCl/EG in the formulation raised its intensity. The peak was associated with C-H_2_ bonds or antisymmetric stretching of C-F_2_ bonds in the β/γ-crystalline phase. The practical disappearance of the peak located at 854 cm^−1^ was also found at concentrations of 1.25 wt% of ChCl/EG, related to the presence of C-H_2_ out of plane rocking and carbon-carbon single bonds (C-C) in phase symmetric stretching for α-crystalline phases. The peak located at a wavelength of 975 cm^−1^ also underwent significant intensity changes with the inclusion of ChCl/EG as an additive. This had significant intensity on the PVDF pristine membrane but decreased as the DES concentration increased, performing so for both the ChCl/EG and the ChCl/GLY. This peak corresponded to C-H_2_ out-of-phase twisting and C-F_2_ bonds in phase symmetric stretching for α-crystalline phases. At 1145 cm^−1^, correlated with the C-F_2_ bonds in phase stretching, the α-crystalline phase suffered a disappearance with DES inclusion, in the same way as the peak located at 1209 cm^−1^ for C-F_2_ in phase antisymmetric stretching. For 1177 and 1383 cm^−1^, both presented a decrease in intensity with increasing ChCl/EG concentration, the first one being associated with C-F_2_ out-of-phase antisymmetric stretching for α, β, or γ phases, and the second one being associated with C-H_2_ out-of-phase wagging and C-C out-of-phase antisymmetric stretching only in α structures. The peak recorded at 1177 cm^−1^ suffered a shift toward wavelengths to 1165 cm^−1^ when the ChCl/EG concentration was increased. In addition, the loss of intensity and the flattening of this peak could be related to the appearance of C-O groups for the inclusion of DES in the dope solution [71]. This may have been due to the formation of C-H_2_ bonds whose stretching intensity displaced the previously recorded C-F_2_ bonds. Similar results to those observed with ChCl/EG were obtained when ChCl/GLY was used as an additive (Figure 2b).

**Figure 3:**
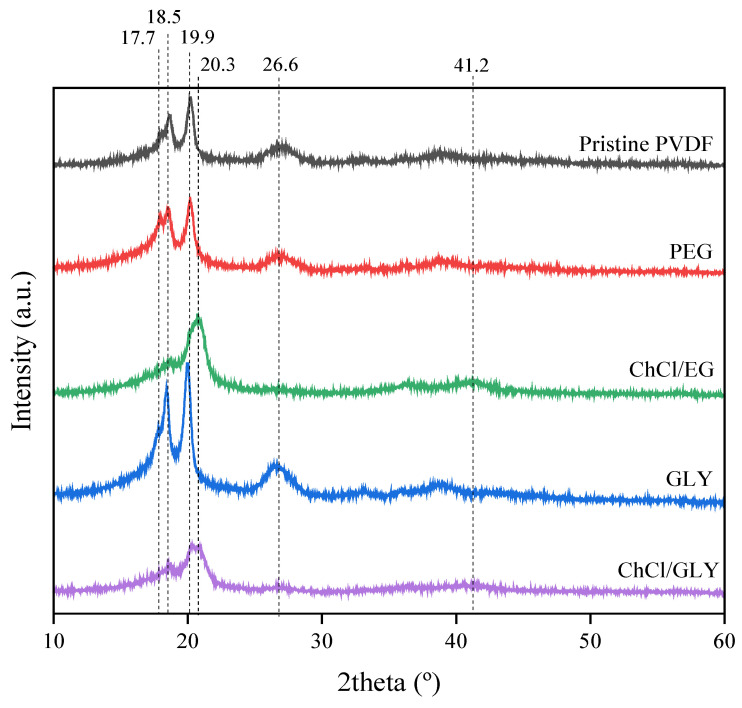
XRD diffractogram of the membranes with the different additives at 5.00 wt%: poly(ethylene glycol) (PEG); choline chloride/ethylene glycol (1:2) DES (ChCl/EG); glycerol (GLY); choline chloride/glycerol (1:2) DES (ChCl/GLY).

XRD analyses were performed to corroborate the crystalline phase changes suggested by the previous FTIR analyses. Figure 3 presents the results collected from the XRD analysis of the PVDF pristine membrane and the membranes prepared with a 5.0 wt% additive concentration. The XRD analyses showed similar diffractograms for the pristine membrane and the membranes with 5.0 wt% of PEG and GLY. Single peaks were located at 17.7, 19.9, and 26.6°, which corresponded to α-crystalline phases of the PVDF [72], along with a peak at 18.5°, attributed to the presence of a γ-crystalline phase, which is present for all membranes at different intensities. However, diffractograms of the membranes with DES showed significant changes associated with the shift in the crystalline phases of the material. Peaks attributed to the α-crystalline phase (17.7, 19.9, and 26.6°) were reduced or even disappeared. Moreover, two peaks located at 20.3 and 41.2°, associated with the β-crystalline phase, were detected only for the membranes with DESs [72,73]. These results, in agreement with the FTIR analyses, indicated that the presence of the DESs (ChCl/EG and ChCl/GLY) in the dope solution provoked the shift of α-crystalline phases towards β-crystalline phases, especially at high additive concentrations.

**Figure 4:**
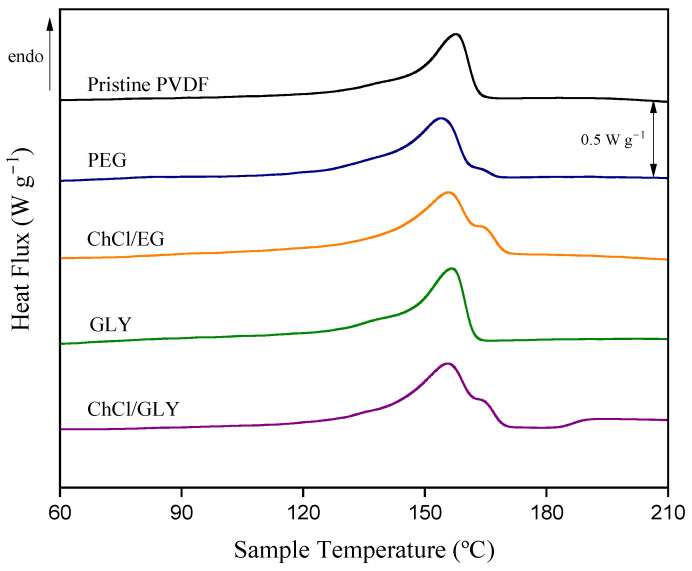
DSC melting process analysis of the membranes with the different additives at 5.0 wt%: poly(ethylene glycol) (PEG); choline chloride/ethylene glycol (1:2) DES (ChCl/EG); glycerol (GLY); choline chloride/glycerol (1:2) DES (ChCl/GLY).

The melting process of PVDF membranes was studied by the comparison of the DSC thermograms of the pristine membrane and membranes with different additives (Figure 4). The presence of PEG in the dope solution resulted in the appearance of a second shoulder close to 165 °C, increasing the total melting curve to 167 °C. The double melting phenomenon could be attributed to different causes [74]: (i) the appearance of a melting, recrystallization, and remelting process during the DSC heating, (ii) the presence of polymorphism, or (iii) the variation of morphology (lamellar thickness, perfection of crystals). Since no β-crystalline phases were observed in the membranes with PEG, the second shoulder in its DSC analysis could be associated with the appearance of a melting, recrystallization, and remelting process. This was observed for all the PEG concentrations tested (Supplementary Material, Figure S4). For the use of ChCl/EG or ChCl/GLY (Figure 4), the shape of the second shoulder changed, increasing its area regarding the membrane with PEG. However, for the case of GLY, the shoulder was not recorded, and the resulting thermograms were similar to those of the pristine PVDF membrane for all the concentrations tested (Figure 4 and Figure S5).

**Figure 5:**
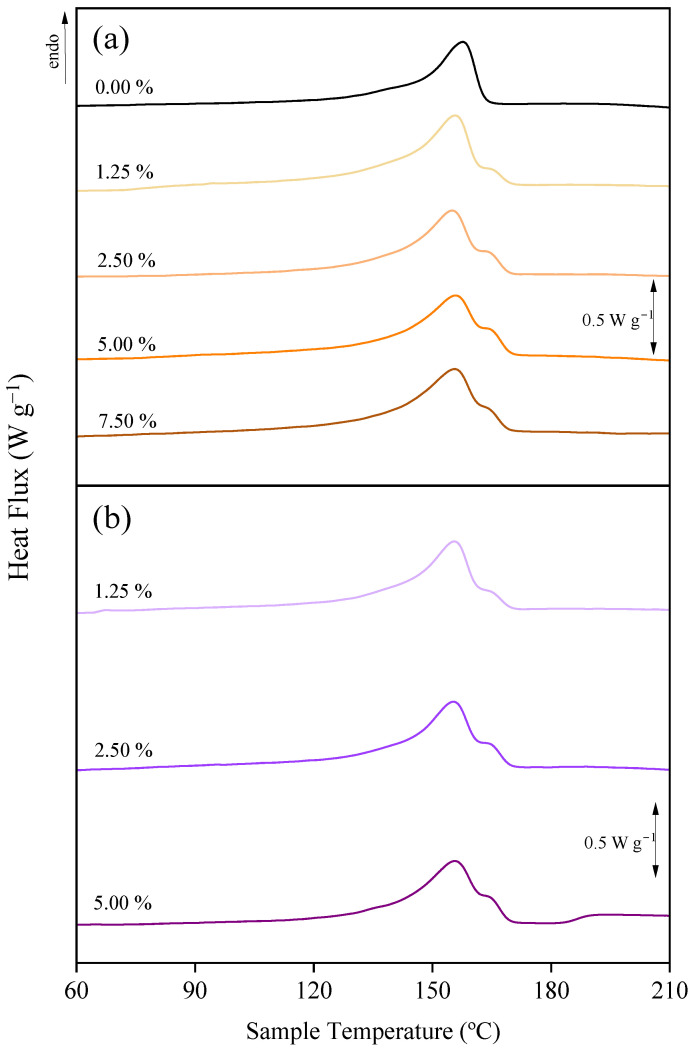
DSC melting process analysis of the pristine PVDF membrane and with different concentrations (wt%) of (**a**) choline chloride/ethylene glycol (1:2) DES (ChCl/EG) and (**b**) choline chloride/glycerol (1:2) DES (ChCl/GLY).

For further analysis of the thermal behavior of the membranes with DES, Figure 5 shows the DSC melting results of the membranes with ChCl/EG and ChCl/GLY. In both cases, an increase in the intensity of the second curve was observed with the increasing additive concentration. Given the increase of β-crystalline phases from FTIR and XRD analysis of these membranes, it was presumed that the increase in the intensity of the second curve was related to the shift of this crystalline phase. In addition, the cooling processes of all membranes produced at different additive concentrations and the pristine membrane were analyzed ( Supplementary Material, Figures S6–S10). However, no significant differences were observed between different additive concentrations or the use of DES versus PEG or GLY.

In Table S1, the melting temperature (T_m_), crystallization temperature (T_c_), and melting enthalpy (Δh_m_) are presented for all the membranes. The pristine PVDF membrane showed its melting temperature (T_m_) at 157.7 °C. Similar results were obtained in PVDF film under different preparation conditions, with a T_m_ of 158.0 °C [69]. The enthalpy results of the DSC analysis are also collected in Table S1, with enthalpy values close to 50 J g^−1^ for all prepared membranes. These values, along with FTIR, were used to estimate the fractions of the PVDF crystalline phases present in each of the membranes, and they were quantified to observe their variation with the different additive concentrations. Considering the minimal presence of γ-phase in the prepared membranes, only α-phase and β-phase were assumed to be present. Thus, Table 5 shows the α-phase fraction according to the intensities measured in the FTIR analysis using Equation (3). The presented results were referred to as the α-phase, with the β-phase being the remaining percentage for 100% crystallinity. As expected, membranes with PEG and GLY did not suffer significant variations in the α-crystalline fraction, regardless of the concentration of such additives contained. Regarding the use of DES, membranes with ChCl/EG and ChCl/GLY resulted in a decrease in the α-crystalline phase compared to the pristine membrane, reaching values close to 45% for a concentration of 1.25 wt%. In the case of higher concentration of the DES, a decrease in the α-crystalline phase in membranes was observed, achieving a value close to 18% for the maximum concentration studied in both cases. The increase in the β-crystalline fraction could be due to the high polarity of the polymeric phase components with the non-solvent, according to Purushothaman et al. (2024) [69]. Thus, it could be related with the increase in additives of a solvent nature, ChCl/EG and ChCl/GLY, accelerating the kinetics of the NIPS process during membrane formation. The degree of crystallinity was calculated, but no significant differences were observed between the different additives used or concentrations, indicating that the material did not lose crystallinity but suffered a change in the balance of α and β phases.

## 4. Conclusions

This study investigated by comprehensive material characterization the impact of various additives on the properties of PVDF membranes using triethyl phosphate (TEP) as a green solvent. Through a combination of FESEM, FTIR, XRD, and DSC analyses, it was observed that additives like poly(ethylene glycol) (PEG), glycerol (GLY), and deep eutectic solvent (DES) compounds based on choline chloride with ethylene glycol (EG) and glycerol (GLY), i.e., ChCl/EG and ChCl/GLY, significantly influenced the membrane’s internal structure, porosity, crystalline phases, and thermal properties. PEG acted as a pore-former, resulting in a transition from a spherulitic to a sponge-like structure, with pore sizes remaining consistent regardless of additive concentration. GLY, similarly, influenced pore formation but introduced nodular morphology at higher concentrations due to viscosity effects on the dope solution. ChCl/GLY resulted in lower membrane pore size than ChCl/EG and GLY. ChCl/EG and ChCl/GLY had a distinct impact, shifting the crystalline phase composition from α to β phases as concentrations increased. This transformation was corroborated by both FTIR and XRD analyses, revealing the emergence of β-crystalline phases to the detriment of α phases, particularly with the DES at higher concentrations. Thermal analysis, using DSC, highlighted the introduction of a second melting curve in DES-modified membranes, correlating with the increased presence of β-crystalline phases, even though the overall crystalline fraction was stable. The high concentrations of certain additives, specifically >10 wt% of ChCl/EG and GLY, as well as ≥7.5 wt% of ChCl/GLY, resulted in membranes with poor mechanical integrity, limiting their characterization. This highlights a key experimental constraint that should be considered in future studies aiming to optimize the formulation and structural stability of PVDF membranes. In summary, the incorporation of the deep eutectic solvents ChCl/EG and ChCl/GLY can help tailor the structural pore shape and distribution, along with the morphological and hydrophobic characteristics of PVDF membranes, offering a versatile approach to prepare membranes for specific applications.

## Figures and Tables

**Table 1:** Viscosity (cP) of the prepared dope solutions of PVDF with triethyl phosphate and different concentrations of additives (wt%).

Additive Conc.	No Additive	PEG *	ChCl/EG *	GLY *	ChCl/GLY *
0.00	8400	-	-	-	-
1.25	-	8200	8200	8000	8700
2.50	-	8500	8400	10,000	8000
5.00	-	11,000	8800	12,000	8500
7.50	-	13,000	10,000	20,000	-
10.00	-	12,500	-	-	-

* PEG: Poly(ethylene glycol); ChCl/EG: choline chloride/ethylene glycol (1:2) DES; GLY: glycerol; ChCl/GLY: choline chloride/glycerol (1:2) DES. Results are within a precision of ±300 cP.

**Table 2:** FESEM images of the surface and cross-section of the PVDF pristine membrane and with additives at different concentrations (wt%): (a) poly(ethylene glycol) (PEG), (b) choline chloride/ethylene glycol (1:2) DES (ChCl/EG), (c) glycerol (GLY), and (d) choline chloride/glycerol (1:2) DES (ChCl/GLY).

	0.00%	1.25%	2.50%	5.00%	7.50%	10.00%
	**(a)** **PEG**					
Surface	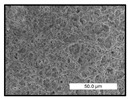	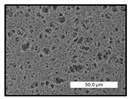	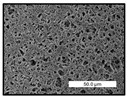	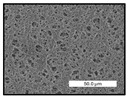	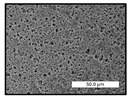	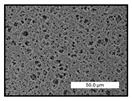
Cross-section	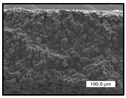	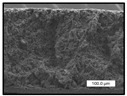	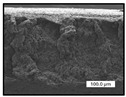	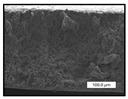	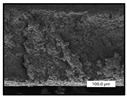	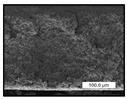
	**(b)** **ChCl/EG**					
Surface	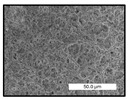	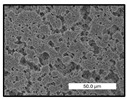	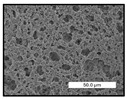	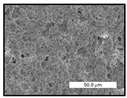	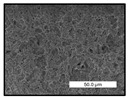	-
Cross-section	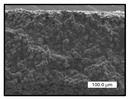	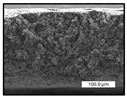	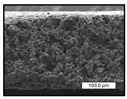	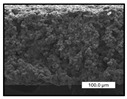	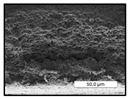	-
	**(c)** **GLY**					
Surface	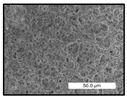	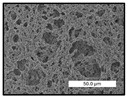	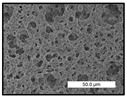	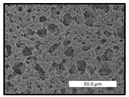	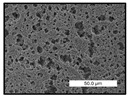	-
Cross-section	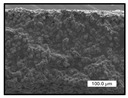	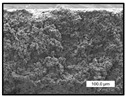	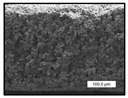	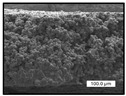	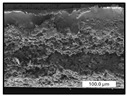	-
	**(d)** **ChCl/GLY**					
Surface	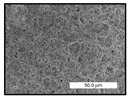	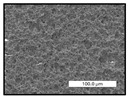	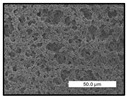	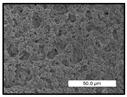	-	-
Cross-section	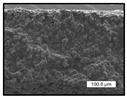	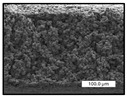	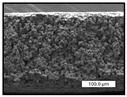	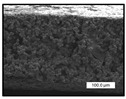	-	-

**Table 3:** Overall porosity, thickness, surface density, and most probable pore size of the fabricated PVDF membranes at different concentrations of additives (wt%).

Additive Conc.	No Additive	PEG *	ChCl/EG *	GLY *	ChCl/GLY *
**Overall porosity (%)**					
0.00	80.4 ± 2.1	-	-		-
1.25	-	81.4 ± 2.4	79.7 ± 2.3	82.2 ± 2.0	80.4 ± 2.3
2.50	-	74.3 ± 0.5	79.1 ± 2.1	84.1 ± 1.1	74.8 ± 0.6
5.00	-	78.4 ± 0.8	75.6 ± 1.1	79.7 ± 1.5	75.0 ± 0.6
7.50	-	83.4 ± 0.9	74.9 ± 0.5	82.4 ± 1.6	-
10.00	-	80.6 ± 2.4	-	-	-
**Thickness (µm)**					
0.00	294.2 ± 16.5	-	-		-
1.25	-	304.4 ± 3.2	242.7 ± 0.1	318.8 ± 15.6	262.6 ± 5.3
2.50	-	290.8 ± 9.9	248.2 ± 4.1	300.6 ± 11.4	245.7 ± 7.5
5.00	-	298.6 ± 8.8	234.1 ± 3.8	305.4 ± 0.1	232.7 ± 7.4
7.50	-	295.3 ± 3.9	244.1 ±7.4	312.8 ±10.3	-
10.00	-	279.7 ± 8.8	-	-	-
**Surface density (mg cm^−2^)**					
0.00	10.2 ± 0.4	-	-	-	-
1.25	-	10.6 ± 0.5	10.1 ± 0.7	10.2 ± 0.3	10.2 ± 0.7
2.50	-	10.1 ± 0.1	10.9 ± 0.4	10.1 ± 0.3	10.0 ± 0.2
5.00	-	10.8 ± 0.6	10.4 ± 0.7	10.5 ± 0.6	10.9 ± 0.5
7.50	-	10.8 ± 0.1	10.3 ± 0.2	10.7 ± 0.3	-
10.00	-	10.0 ± 0.1	-	-	-
**Most probable pore size, D_p_ (µm)**					
0.00	3.30 ^b^	-	-	-	-
1.25	-	0.77 ^a^	3.85 ^b^	2.31 ^b^	2.10 ^b^
2.50	-	0.66 ^a^	5.78 ^c^	3.85 ^b^	2.72 ^b^
5.00	-	0.77 ^a^	5.78 ^c^	3.55 ^b^	>d.l.
7.50	-	0.77 ^a^	>d.l.	2.01 ^b^	-
10.00	-	0.71 ^a^	-	-	-

* PEG: Poly(ethylene glycol); ChCl/EG: choline chloride/ethylene glycol (1:2) DES; GLY: glycerol; ChCl/GLY: choline chloride/glycerol (1:2) DES; d.l.: detection limit = 6.60 µm. ^a, b, c^ standard deviation of pore size difference related to pressure variation; ^a^ (range < 2 µm) → 0.05 µm; ^b^ (range [2–4] µm) → 0.07 µm; ^c^ (range > 4 µm) → 0.19 µm.

**Table 4:** Water contact angle (°) of the fabricated PVDF membranes at different concentrations of additives (wt%).

Additive Conc.	No Additive	PEG *	ChCl/EG *	GLY *	ChCl/GLY *
0.00	143.9 ± 2.2	-	-	-	-
1.25	-	128.2 ± 4.2	130.9 ± 4.8	133.5 ± 5.6	138.9 ± 4.1
2.50	-	130.3 ± 4.3	145.4 ± 2.0	132.0 ± 4.1	135.1 ± 3.1
5.00	-	129.1 ± 1.9	145.7 ± 1.6	136.0 ± 2.7	146.8 ± 2.8
7.50	-	118.9 ± 2.0	127.3 ± 4.1	101.8 ± 5.3	-
10.00	-	118.1 ± 5.7	-	-	-

* PEG: Poly(ethylene glycol); ChCl/EG: choline chloride/ethylene glycol (1:2) DES; GLY: glycerol; ChCl/GLY: choline chloride/glycerol (1:2) DES.

**Table 5:** (a) Quantification of α-crystalline phase (%) and (b) crystallinity degree (%) of the PVDF membranes with different concentrations of additives (wt%).

Additive Conc.	No Additive	PEG *	ChCl/EG *	GLY *	ChCl/GLY *
**(a) α-crystalline phase (%)**					
0.00	75.0	-	-	-	-
1.25	-	75.4	48.0	76.5	44.0
2.50	-	75.6	24.3	74.8	39.2
5.00	-	72.8	19.2	76.1	18.2
7.50	-	73.4	18.1	77.2	-
10.00	-	72.6	-	-	-
**(b) Crystallinity degree (%)**					
0.00	53.1	-	-	-	-
1.25	-	51.7	53.7	56.0	55.5
2.50	-	54.2	51.1	51.7	51.6
5.00	-	47.5	51.3	51.6	50.8
7.50	-	47.1	49.7	54.4	-
10.00	-	50.5	-	-	-

* PEG: Poly(ethylene glycol); ChCl/EG: choline chloride/ethylene glycol (1:2) DES; GLY: glycerol; ChCl/GLY: Choline chloride/glycerol (1:2) DES.

## Data Availability

Data will be available on Zenodo: https://doi.org/10.5281/zenodo.15131619.

## Supplementary Materials

The following supporting information can be downloaded at https://www.mdpi.com/article/10.3390/polym17070984/s1.

## Abbreviations

(Δh)_m_: J g^−1^	melting enthalpy
ChCl	choline chloride
ChCl/EG	choline chloride/ethylene glycol (1:2) DES
ChCl/GLY	choline chloride/glycerol (1:2) DES
DES	deep eutectic solvent
DSC	differential scanning calorimetry
EG	ethylene glycol
FESEM	field emission scanning electron microscopy
FTIR	Fourier transform-infrared spectroscopy
GLY	glycerol
HBA	hydrogen bond acceptor
HBD	hydrogen bond donor
NIPS	non-solvent induced phase separation
OcOH	1-octanol
PEG	poly(ethylene glycol)
PVDF	poly(vinylidene fluoride)
D_p_, µm	pore size distribution
T_c_, °C	crystallization temperature
TEP	triethyl phosphate
T_m_, °C	melting temperature
WCA, °	water contact angle
x	α-crystalline phase
X, %	degree of crystallinity
XRD	X-ray diffraction
y	β-crystalline phase
