# Supplementary material for: Effect of Choline Chloride-Based DES on the Pore-Forming Ability and Properties of PVDF Membranes Prepared with Triethyl Phosphate as Green Solvent
Source: Polymers (Basel). 2025 Apr 4;17(7):984. doi: 10.3390/polym17070984 (PMC11991192; doi:10.3390/polym17070984)
Supplement: Supplementary file 1 [file polymers-17-00984-s001.zip › polymers-3544669-supplementary.pdf]

Supplementary Material for

Effect of Choline Chloride-Based DES on the Pore-Forming Ability and Properties of PVDF Membranes Prepared with Triethyl Phosphate as Green Solvent

Alejandro Gálvez-Subiela, Ramón Jiménez-Robles, Jose David Badia-Valiente, Marta Izquierdo * and Amparo Chafer *

Research Group in Materials Technology and Sustainability (MATS), Department of Chemical Engineering, School of Engineering, University of Valencia, Avda. Universitat s/n, 46100 Burjassot, Spain; alejandro.galvez@uv.es (A.G.-S.); ramon.jimenez@uv.es (R.J.-R.); jose.badia@uv.es (J.D.B.-V.)

* Correspondence: marta.izquierdo-sanchis@uv.es (M.I.); amparo.chafer@uv.es (A.C.)

S1. Infrared spectra (FTIR) of the additives used and PVDF membranes with poly (ethylene glycol) and glycerol as additives.

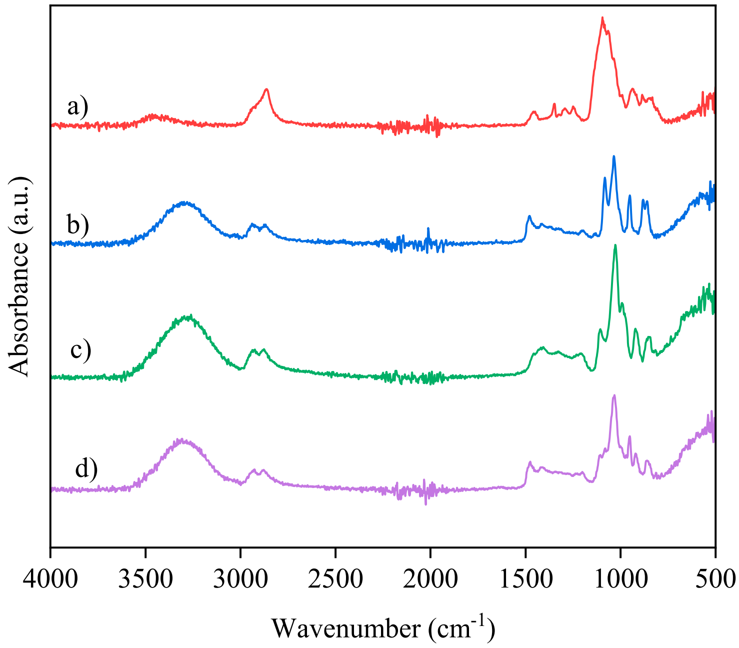

Figure S1. FTIR spectra of the additives employed: a) poly (ethylene glycol) (PEG), b) choline chloride: ethylene glycol (1:2) DES (ChCl:EG), c) glycerol (GLY) and, d) choline chloride: glycerol (1:2) DES (ChCl:GLY).

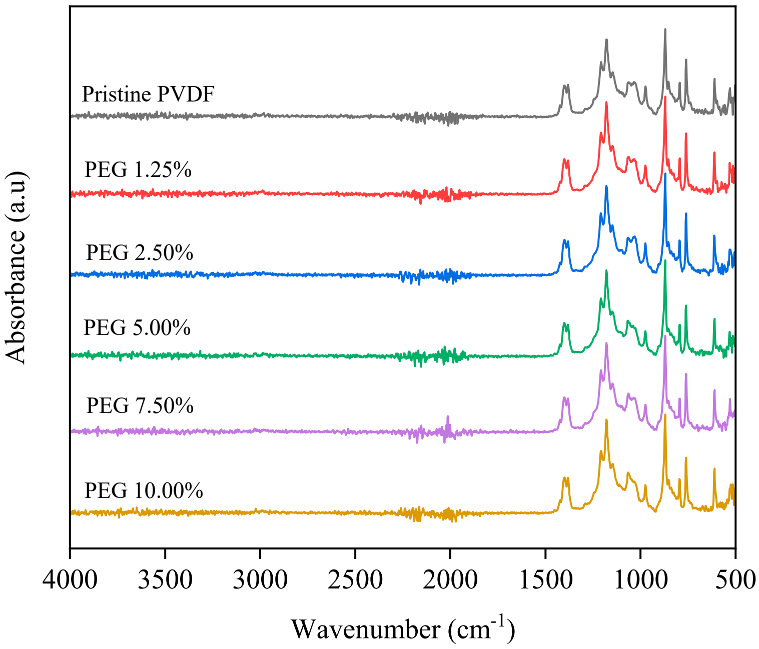

Figure S2. FTIR spectra of the membranes with different poly (ethylene glycol) (PEG) concentrations (wt%).

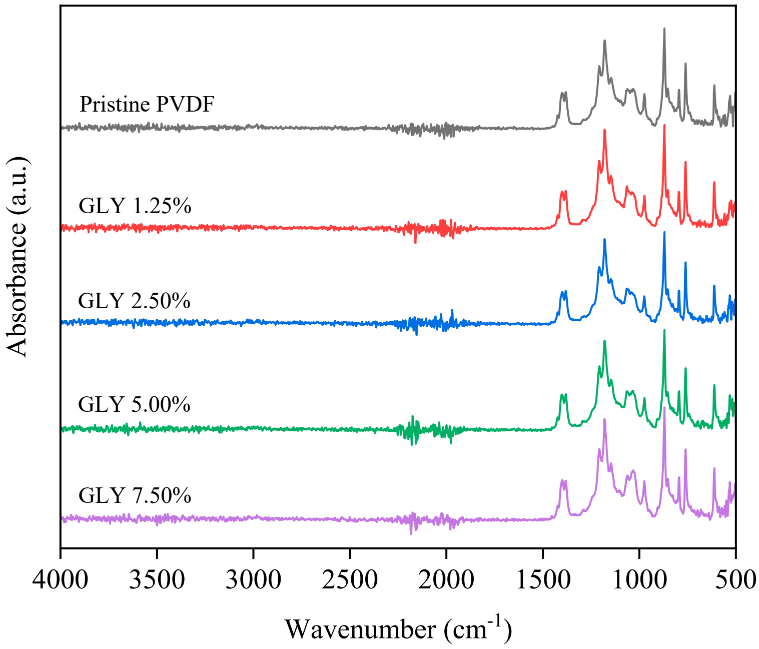

Figure S3. FTIR spectra of the membranes with different glycerol (GLY) concentrations (wt%).

S2. Differential scanning calorimetry (DSC) for melting and cooling processes of the pristine PVDF membrane and additives studied.

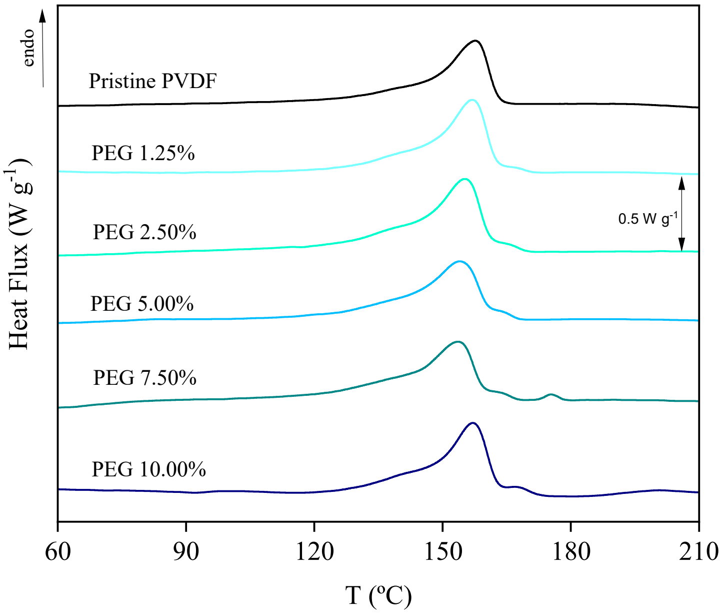

Figure S4. DSC melting process analysis of the membranes with different poly (ethylene glycol) (PEG) concentrations (wt%).

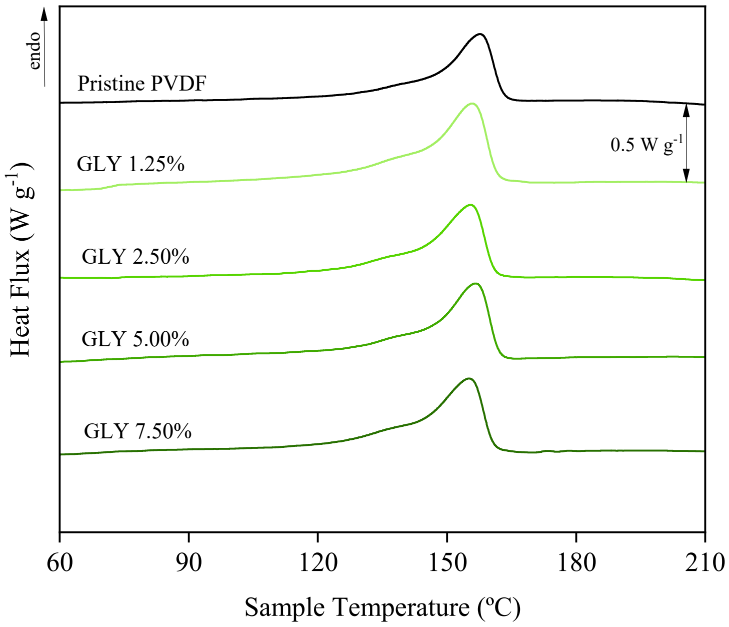

Figure S5. DSC melting process analysis of the membranes with different glycerol (GLY) concentrations (wt%).

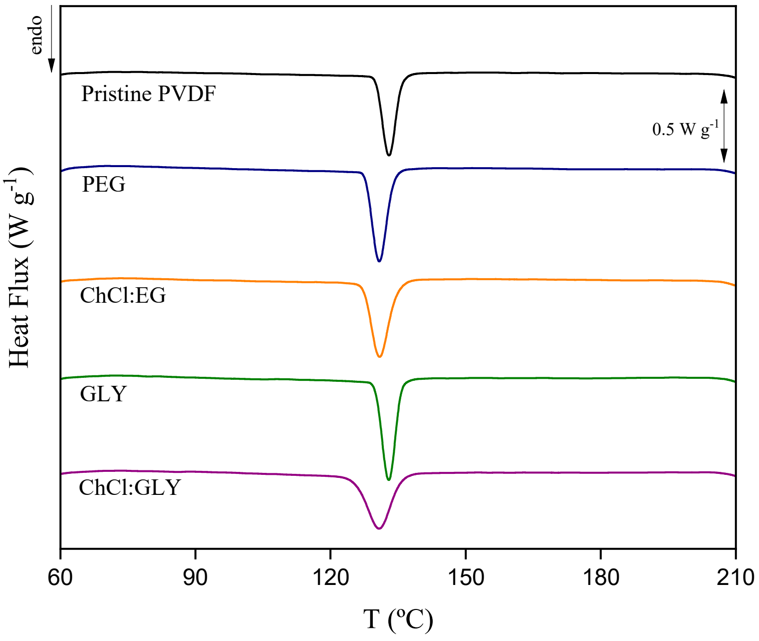

Figure S6. DSC cooling scan of the elaborated membranes with the different additives at 5.00 wt%: poly (ethylene glycol) (PEG); choline chloride: ethylene glycol (1:2) DES (ChCl:EG); glycerol (GLY); choline chloride: glycerol (1:2) DES (ChCl:GLY).

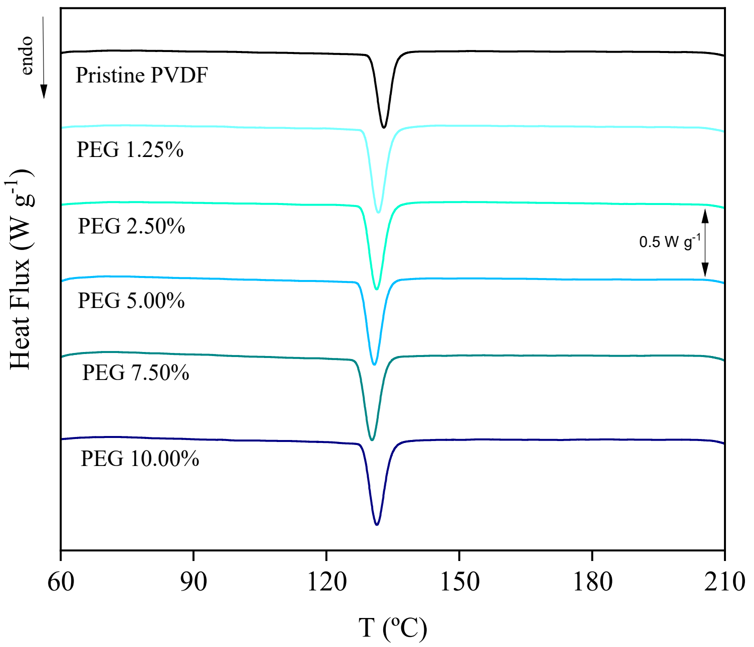

Figure S7. DSC cooling scan of the elaborated membranes with different poly (ethylene glycol) (PEG) concentrations (wt%).

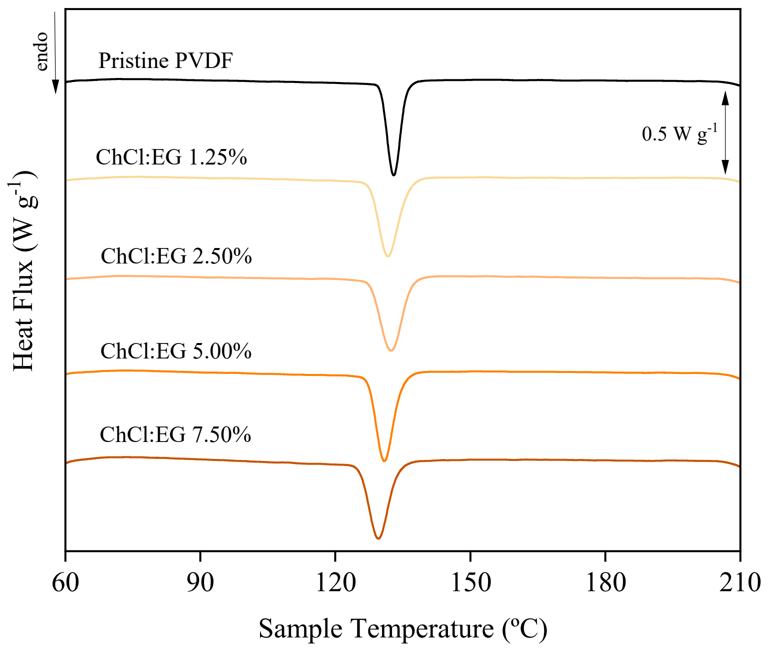

Figure S8. DSC cooling scan of the elaborated membranes with different choline chloride: ethylene glycol (1:2) DES (ChCl:EG) concentrations (wt%).

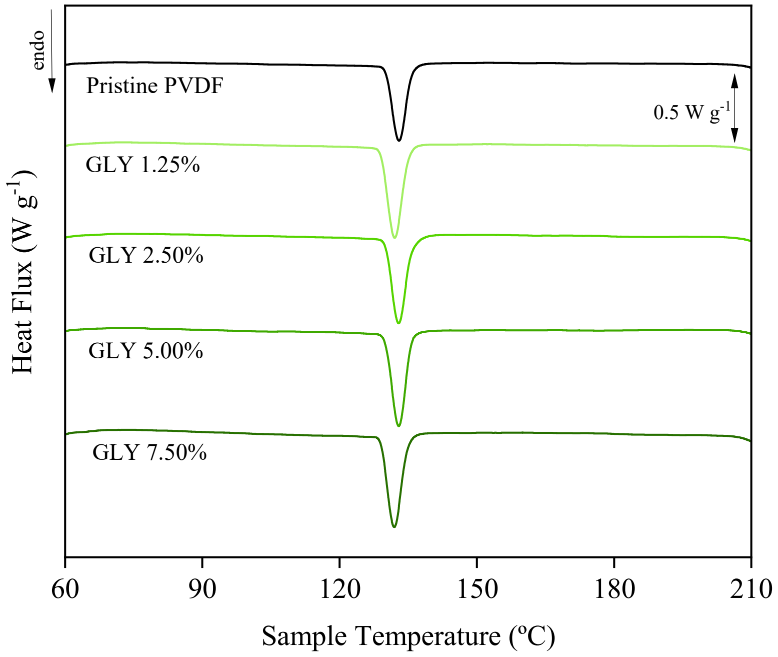

Figure S9. DSC cooling scan of the elaborated membranes with different glycerol (GLY) concentrations (wt%).

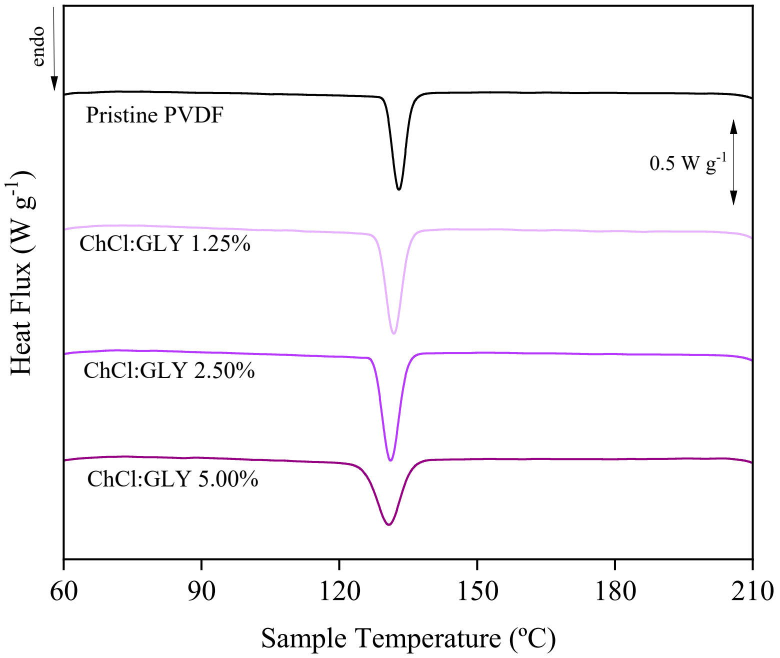

Figure S10. DSC cooling scan of the elaborated membranes with different choline chloride: glycerol (1:2) DES (ChCl:GLY) concentrations (wt%).

S3. Melting temperature, crystallization temperature and enthalpies of the elaborated membranes.

Table S1. Melting temperatures (T_m), crystallization temperatures (T_c), and enthalpies (Δh_m) of the elaborated membranes with different concentrations of additives (wt%).

	**Additive conc.**	**T_m (°C) (main peak)**	**T_c (°C)**	**Δh_m (J g^{-1})**
**Pristine PVDF**	0.00	157.7	133.0	50.8 ± 0.8
**PEG***	1.25	156.9	131.8	49.4 ± 0.8
	2.50	155.3	131.3	51.8 ± 1.6
	5.00	154.0	130.8	45.5 ± 0.0
	7.50	153.6	130.3	45.1 ± 1.6
	10.00	157.1	131.4	48.4 ± 4.2
**ChCl:EG***	1.25	155.8	131.7	52.8 ± 2.4
	2.50	155.0	132.4	51.6 ± 0.7
	5.00	155.9	130.9	52.0 ± 1.8
	7.50	155.6	129.6	50.4 ± 0.1
**GLY***	1.25	155.9	132.0	53.4 ± 0.7
	2.50	155.6	132.9	49.5 ± 0.6
	5.00	156.7	132.9	49.3 ± 3.4
	7.50	155.2	132.0	51.9 ± 1.8
**ChCl:GLY***	1.25	155.6	131.9	54.9 ± 2.3
	2.50	155.3	131.2	51.2 ± 3.3
	5.00	155.7	130.8	51.6 ± 0.8

*PEG: Poly (ethylene glycol); ChCl:EG: Choline chloride: ethylene glycol (1:2) DES; GLY: Glycerol; ChCl:GLY: Choline chloride: glycerol (1:2) DES.
